# Pre-Test Probability Assessment and d-Dimer Based Evaluation in Patients with Previous Acute Aortic Syndrome

**DOI:** 10.3390/medicina59030548

**Published:** 2023-03-10

**Authors:** Fulvio Morello, Marco Santoro, Francesca Giachino, Francesca Caciolli, Elisa Capretti, Matteo Castelli, Emanuele Pivetta, Peiman Nazerian, Enrico Lupia

**Affiliations:** 1 S.C. Medicina d’Urgenza U (MECAU), Ospedale Molinette, A.O.U. Città della Salute e della Scienza, 10126 Torino, Italy; 2 Dipartimento di Scienze Mediche, Università degli Studi di Torino, 10126 Torino, Italy; 3 Dipartimento di Emergenza e Accettazione, A.O.U. Careggi, 50134 Firenze, Italy

**Keywords:** acute aortic syndrome, aortic dissection, clinical score, d-dimer, diagnosis

## Abstract

*Background and Objectives*. Acute aortic syndromes (AASs) are emergencies burdened by high morbidity and mortality. Guideline-recommended diagnostic workup is based on pre-test probability assessment (PPA) and d-dimer testing. However, the performance of PPA and d-dimer has never been studied in individuals with previous AAS (pAAS), which represent a challenging population. *Materials and Methods*. We analyzed a registry of patients with pAAS evaluated in two Emergency Departments (EDs) for suspected novel AAS (nAAS). Enrolment criteria were history of pAAS and the presence of truncal pain, syncope or perfusion deficit. All patients underwent advanced imaging. Clinical data were registered prospectively and PPA was performed by applying the aortic dissection detection (ADD) and an aorta simplified (AORTAs) score. *Results*. A total of 128 patients were enrolled, including 77 patients with previous Stanford type A aortic dissection and 45 patients with previous Stanford type B aortic dissection. The final diagnosis was nAAS in 40 (31%) patients. Clinical variables associated with nAAS were: aortic valve disease, thoracic aortic aneurysm, severe pain, sudden pain, ripping/tearing pain and hypotension/shock. ADD score ≥ 2 had a sensitivity of 65% and a specificity of 83% for nAAS; AORTAs score ≥ 2 had a sensitivity of 48% and a specificity of 88%. d-dimer (cutoff ≥ 500 ng/mL or age-adjusted cutoff) had a sensitivity of 97% and a specificity of 13%/14.7%, for diagnosis of nAAS. Patients that were candidates for guideline-compliant PPA/d-dimer integrated rule-out were: 5 (4.9%) with ADD ≤ 1/d-dimer and 8 (7.8%) with AORTAs ≤ 1/d-dimer < age-adjusted cutoff. None of them had a nAAS. *Conclusions*. Patients with pAAS evaluated in the ED for red-flag symptoms showed intermediate-to-high pre-test probability of nAAS. The ADD score had lower sensitivity and specificity than in unselected patients. d-dimer, alone and integrated with PPA, was highly sensitive for nAAS, but very unspecific. PPA/d-dimer integrated strategies are unlikely to significantly reduce the number of patients with pAAS undergoing advanced imaging.

## 1. Introduction

Acute aortic syndromes (AASs), including aortic dissection (AD), intramural aortic hematoma (IMH), penetrating aortic ulcer (PAU) and aortic rupture, are cardiovascular emergencies affecting ≈6–8 cases/100,000 individuals/year [1]. Patients with these syndromes are burdened with severe morbidity and mortality, but rapid diagnosis and transfer to specialized centers positively affects outcomes. The most common symptoms of AASs leading to Emergency Department (ED) evaluation are truncal pain and syncope [2]. However, the clinical spectrum of AASs is variable and highly unspecific. This leads to concurrency of both high misdiagnosis rate and overuse of computed tomography angiography (CTA) [3,4,5]. 

Patients with previous AAS (pAAS) are at risk of developing novel AAS (nAAS) in their lifetime, and suffer increased morbidity and mortality [6,7,8]. nAASs can be more heterogeneous than in aorta-naïve patients. They include, in addition to de novo AASs, acute worsening of chronic aortic disease (e.g., false lumen extension, aortic dilatation with impending rupture), and treatment-related complications such as tube–graft dehiscence and endoleak. 

To standardize clinical assessment and selection of patients necessitating CTA for suspected AAS, guidelines recommend a diagnostic pipeline integrating pre-test probability assessment (PPA) and d-dimer assay [9,10]. PPA stratifies the clinical probability of AAS in a given patient. Validated scores recommended by guidelines for standardized PPA are the aortic dissection detection (ADD) risk score, and the aorta simplified (AORTAs) score, respectively assessing 12 and 6 items of clinical presentation and predisposing conditions [11,12,13]. Since d-dimer is a circulating biomarker with high sensitivity for AASs, low levels of d -dimer associated with low pre-test probability can be used to rule out AASs, without CTA [14,15].

In the last decade, increasing incidence and survival of AASs have led to growing numbers of ED visits for patients with pAAS [16,17,18]. However, the performance of PPA has never been evaluated in this challenging patient subgroup. Furthermore, different studies have reported increased levels of d-dimer in patients with pAAS, especially with chronic aortic dissection, potentially affecting specificity [19,20,21]. Hence, evidence is lacking regarding accuracy and efficiency of a PPA/d-dimer diagnostic bundle in patients with pAAS. In order to fill this gap in knowledge, we evaluated the performance of a PPA/d-dimer strategy in a registry of patients with pAAS, evaluated in the ED for acute symptoms potentially prompting nAAS.

## 2. Materials and Methods

### 2.1. Study Design and Setting

This was an observational study performed in two EDs, located in a tertiary university hospital functioning as regional hub for AASs. The study protocol conformed to the ethical guidelines of the 1975 Declaration of Helsinki, and local Ethics Committees approved the study. Informed consent was obtained from all subjects involved in the study.

### 2.2. Patient Selection

From 2009 to 2016 and from 2019 to 2022, patients with pAAS were enrolled in a registry if they were evaluated in the ED for potential symptoms of nAAS and if they underwent advanced aortic imaging based on clinical suspicion. Red flag symptoms were chest/back/abdominal pain, syncope and signs/symptoms of organ perfusion deficit (i.e., neurological deficit, myocardial ischemia or limb ischemia) dating ≤14 days. Exclusion criteria were: age < 18 years, primary trauma or presence of an obvious alternative diagnosis to nAAS. Enrolment followed convenience sampling and not consecutive enrolment, owing to constraints due to clinical activity.

### 2.3. Advanced Imaging

All patients included in the study underwent contrast-enhanced chest and abdomen computed tomography angiography for conclusive diagnosis. CTA was performed with Lightspeed VCT 64 (GE, Piscataway, NJ, USA) or with Somatom Definition As4 and AS128 (Siemens, Erlangen, Germany). Images were interpreted by a radiologist who was an expert in aortic imaging and, if clinically necessary, by cardiac or vascular surgeons. None of these physicians were involved in the present study.

### 2.4. d-Dimer Assay

Patients underwent venipuncture as part of the initial diagnostic workup and blood samples were immediately sent to the local laboratory for d-dimer assay. Attending physicians were not blinded to test results. During the study, the cutoff used in the study centers was 500 ng/mL. d-dimer levels were measured with an automated latex agglutination test (STA LIATEST^®^ D-DI, DiagnosticaStago, Mannheim Germany, or Hemosil D-dimer HS, Bedford, UK). Laboratory technicians were unaware of the clinical data. 

### 2.5. Pretest Probability Assessment

Clinical data, including variables used for PPA, were acquired and recorded by the treating physician or a researcher during the index visit. During data analysis, PPA was performed by applying the ADD risk score and the AORTAs score, per guidelines [9,10]. 

The ADD score evaluates 12 clinical variables classified in 3 groups: predisposing conditions (Marfan syndrome/other connective tissue disease, family history of aortic disease, known aortic valve disease, known thoracic aortic aneurysm, aortic manipulation in the last month), pain characteristics (sudden pain, severe pain, ripping/tearing pain), and clinical findings (pulse asymmetry/systolic blood pressure differential >20 mmHg, focal neurological deficit, new murmur of aortic regurgitation, shock state/hypotension) [22]. Severe pain was defined by a numeric rating scale ≥7 out of 10, and hypotension as systolic blood pressure ≤90 mmHg. The ADD score corresponds to the number of categories (0 to 3) in which the patient meets at least 1 criterion. The AORTAs score is a simplified score based on 6 clinical variables (thoracic aortic aneurysm, severe pain, sudden pain, pulse deficit, neurologic deficit, shock/hypotension), assigning 2 points to hypotension/shock and 1 to the other items, ranging from 0 to 7 [13]. Based on available studies and guidelines, the pre-test probability was considered low for patients with ADD or AORTAs score ≤1, and high with ADD or AORTAs score ≥2.

### 2.6. Final Adjudication

Two senior physicians established the final diagnosis in each study patient after reviewing all aortic imaging, ED/hospital charts and follow-up data. They were blinded to d-dimer levels. The following conditions were considered as forms of nAAS: de novo AD/IMH/PAU, extension of a chronic false lumen, aortic dilatation, impending aortic rupture, and aortic tube graft-related complications (dehiscence, endoleak).

### 2.7. Statistical Analysis

Continuous variables are presented as median and interquartile range, and categorical variables as absolute number and 95% confidence interval (CI). Continuous variables were compared using non-parametric Mann–Whitney U-test, and categorical variables were compared using χ^2^ test. Diagnostic accuracy was evaluated using receiver operating curve (ROC) analysis. Comparison between area under the curve (AUC) values was performed according to DeLong et al. The clinical usefulness of diagnostic strategies was evaluated using decision curve analysis [23]. *p*-values < 0.05 were considered statistically significant. Statistical analysis was performed with Medcalc ver. 20.115 (MedCalc Software Ltd, Ostend, Belgium) and IBM SPSS Statistics ver. 28.0.1.0 (IBM Corp., Armonk, NY, USA).

## 3. Results

### 3.1. Study Population

We analyzed 128 patients with pAAS evaluated in the ED for suspected nAAS. The pAAS type were: Stanford type A-AD in 77 (60%) patients, Stanford type B-AD in 45 (35%), Stanford type A-IMH in 4 (3%), Stanford type B-PAU in 1 (1%) and aortic rupture in 1 (1%). The demographic and clinical characteristics of study patients are summarized in Table 1. The most common symptom was anterior chest pain.

The final diagnosis was nAAS in 40 (31%) patients and alternative diagnosis (AltD) in 88 (69%). nAAS types were: de novo AAS in 14 (including 7 type A-AD, 6 type B-AD, and 1 IMH; 35% of nAASs) patients, chronic false lumen extension in 7 (18%), aortic dilatation in 4 (10%), prosthetic complications in 7 (18%) and aortic rupture in 8 (20%). In patients with AltD, the final diagnoses were: muscle–skeletal pain in 26 (20% of AltDs) patients, gastrointestinal disease in 11 (9%), acute coronary syndrome in 3 (2%), syncope in 3 (2%), pneumonia in 1 (1%), stroke in 3 (2%) and other/unidentified diagnosis in 39 (30%). 

Patients with nAAS had lower diastolic blood pressure, increased white blood cell count and increased C-reactive protein compared to patients with AltDs.

### 3.2. Pretest Probability Assessment

Table 2 summarizes the prevalence of clinical variables used for PPA and their associated odds ratio (OR). Variables statistically associated with a diagnosis of nAAS were: known aortic valve disease, known thoracic aortic aneurysm, severe pain, sudden pain, ripping/tearing pain and hypotension/shock state. Most patients were classified at low pre-test probability of AAS (i.e., ADD or AORTAs score ≤ 1). The prevalence of nAASs was 17.6% in patients with ADD score = 0, 16.1% in patients with ADD score ≤ 1, 63.4% in patients with ADD score ≥ 2, 21.4% in patients with AORTAs score ≤ 1 and 63.3% in patients with an AORTAs score ≥ 2.

**Figure 1:**
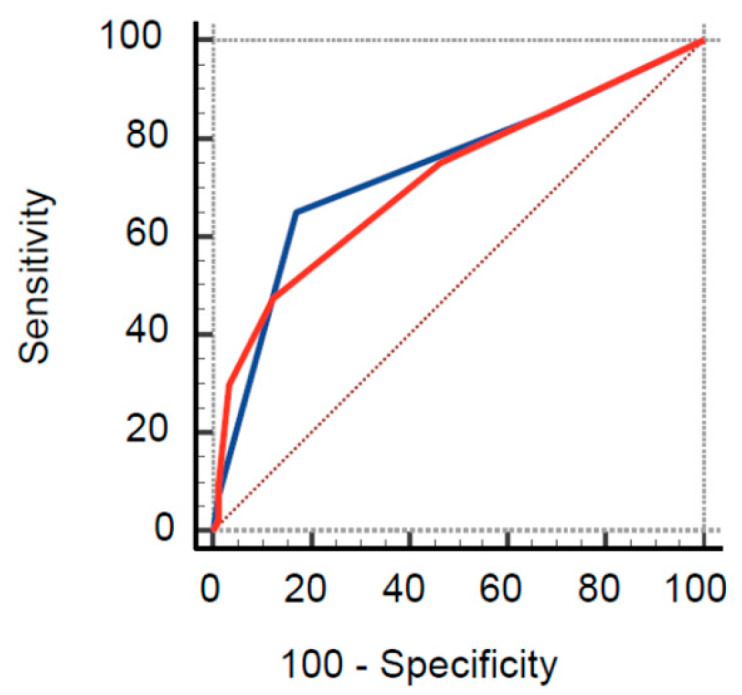
ROC curve of ADD (blue line) and AORTAs (red line) score for diagnosis of novel acute aortic syndrome.

On ROC analysis, the AUC for the ADD and AORTAs score were 0.74 (95% CI 0.65–0.81) and 0.72 (95% CI 0.63–0.79), respectively (Figure 1; *p* = 0.55). 

The diagnostic variables of the clinical scores for diagnosis of nAAS are shown in Table 3. ADD ≥ 1 provided the highest sensitivity, with the lowest specificity.

### 3.3. d-Dimer

**Figure 2:**
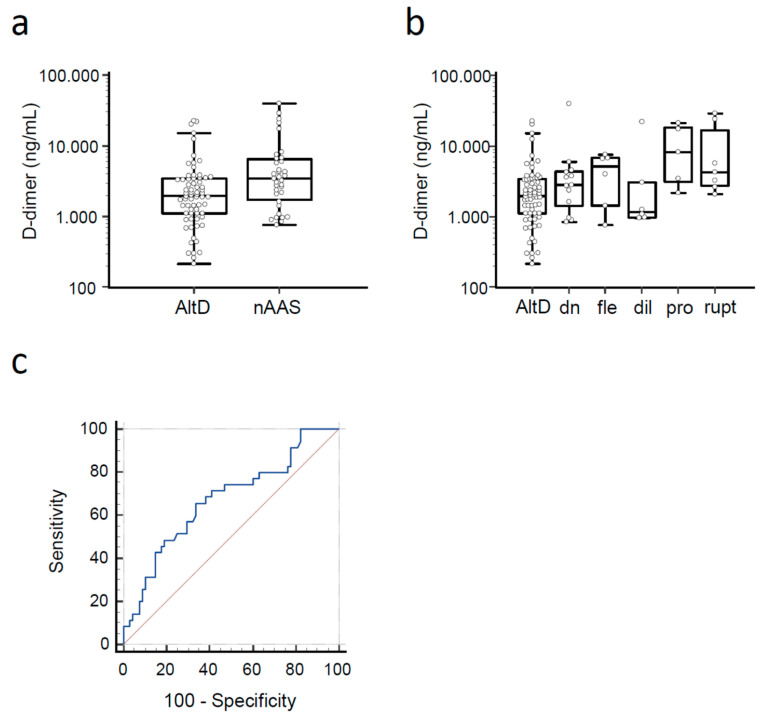
(**a**) d-dimer levels in study patients with novel acute aortic syndrome (nAAS) and alternative diagnosis (AltD). (**b**) d-dimer levels in study patients with nAAS, classified by disease subtype. dn: de novo acute aortic syndrome; fle: false lumen extension; dil: aortic dilatation; pro: prosthetic complication; rupt: aortic rupture. (**c**) ROC curve of d-dimer for diagnosis of novel acute aortic syndrome.

A d -dimer test result was available for 103 (80.5%) patients. Median d-dimer levels were 3480 (IQR 1750–6512) ng/mL in patients with nAAS and 1975 (IQR 1110–3456) ng/mL in patients with AltD (*p* = 0.004, Figure 2a). d-dimer levels in different nAAS subtypes are shown in Figure 2b. The AUC of d-dimer for diagnosis of nAAS was 0.67 (95% CI 0.57–0.76; Figure 2c). 

The diagnostic variables of d-dimer for diagnosis of nAAS, associated with different cutoff values, are shown in Table 4.

### 3.4. Integrated Pathway

Patients who were candidates for current guideline-compliant PPA/d-dimer integrated rule-out were: 5 (4.9%) with ADD ≤ 1/d-dimer < 500 ng/mL, and 8 (7.8%) with AORTAs ≤ 1/d-dimer < age-adjusted cutoff. None of them had a nAAS. The diagnostic performance variables of different rule-out strategies are shown in Table 5. 

**Figure 3:**
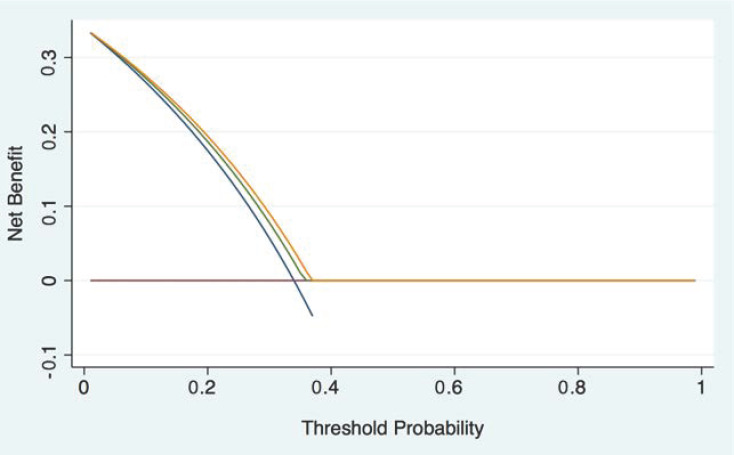
Decision curve analysis of guideline-compliant integrated rule-out rules. Blue line: treat all; red line: treat none; green line: ADD ≤ 1/d-dimer <500 ng/mL; orange line: AORTAs ≤ 1/d-dimer < age-adjusted cutoff.

DCA shows that PPA/d-dimer rule-out strategies have marginal clinical usefulness, compared to the hypothesis of considering all study patients as having a nASS (Figure 3).

## 4. Discussion

A key finding of the present study is that, in patients with pAAS evaluated in the ED for truncal pain and other red flag symptoms, the rate of nAAS was substantial (28%). In previous ED studies from our group performed on unselected patients with similar symptoms, the prevalence of AASs was 13% to 22% [12,24,25]. In these cohorts, the prevalence of AASs was 2.7–5.9% in patients with ADD score = 0 (low risk), 9–27.3% in patients with ADD score = 1 (intermediate risk), and 39% in patients with ADD score ≥ 2 (high risk). In the current study focusing on patients with pAAS, the prevalence of nAAS was 17.6% in individuals with an ADD score = 0. These results indicate that a history of pAAS constitutes a major risk factor for nAAS, defining intermediate-to-high pre-test probability of nAAS.

Study patients with nAAS, compared to unselected patients from the IRAD database, showed lower prevalence of anterior chest pain (52% vs. 61%), sudden pain (43% vs. 79%) and severe pain (40% vs. 91%). These findings indicate that the clinical picture of nAAS may be subtler, rendering the diagnosis even more elusive than in primary AAS. However, the proportion of patients with nAAS presenting as critical (shock, neurological deficit) was unchanged. In patients with pAAS, the overall accuracy of clinical scores used for PPA was similar to previous estimates in unselected patients [13]. However, the sensitivity and the specificity of the ADD score in patients with pAAS (85% and 31% for ADD ≥ 1) were lower than those reported (90% and 40%) in unselected patients.

d-dimer is highly sensitive and poorly specific for the diagnosis of AAS [26]. Elevated levels of d-dimer have been previously reported in patients with chronic aortic dissection, indicating persisting thrombotic burden within the false lumen, and have been related to disease progression [19,20,21]. In line with these findings, we found increased levels of d-dimer in most patients with pAAS, further increasing with nAAS. nAAS was also associated with higher concentrations of white blood cells and C-reactive protein, supporting previous evidence that coagulation and inflammation are intertwined processes in AASs [27]. Accordingly, the sensitivity of d-dimer for nAAS was very high, but the specificity was negligible. These findings implicate that low levels of d-dimer strongly argue against nAAS in patients with pAAS and support an alternative diagnosis. However, a very limited proportion of patients are likely to benefit from this approach, as compared to AAS-naïve patients. Comparison of d-dimer levels with previous serial measurements of d-dimer might help to distinguish acute events from basal unspecific elevation, but these data are typically not available in the ED. 

The current study has limitations. A key limit is represented by clinical heterogeneity of patients with pAAS and nASS in terms of disease subtype, anatomical location, previous treatments (medical, endovascular, surgical) and complications, and false lumen patency. This heterogeneity is likely to affect the incidence (i.e., the probability) of nAAS, the levels of d-dimer and the overall accuracy of diagnostic algorithms. However, systematic and detailed collection of these data was not planned in this study, and due to the rarity of these conditions, the study is underpowered for subgroup analysis.

## 5. Conclusions

In conclusion, we found that in patients with pAAS and nAAS, anterior chest pain, sudden and severe pain were less prevalent than in unselected patients with AAS. pAAS was associated with an intermediate-high pre-test probability of nAAS, indicating that pAAS warrants a high index of clinical suspicion for nAAS. d-dimer retained optimal sensitivity for nAAS, but showed very low specificity, limiting rule-out efficiency. Results implicate that in patients with pAAS at low probability based on ADD or AORTAs score, testing negative for d-dimer, CTA could be safely avoided. However, PPA/d-dimer based strategies are expected to only marginally reduce the number of patients requiring CTA for conclusive diagnosis. These strategies may be considered essentially in patients at low PPA in whom CTA is more cumbersome, e.g., in individuals with a history of allergy, renal failure, or frequent CTA use leading to substantial radiation. Taken together, in patients with pAAS evaluated in the ED, presence of red flag symptoms should almost invariably lead to CTA for conclusive diagnosis, and a d-dimer assay should never delay imaging if clinical suspicion is meaningful. 

## Figures and Tables

**Table 1:** Demographic and clinical characteristics of study patients.

	All Patients (*n* = 128)	nAAS (*n* = 40)	AltD (*n* = 88)	*p*-Value
Age (years), median (IQR)	66 (57–75)	63 (55–75)	68 (57–74)	0.58
Gender (female), *n* (%)	33 (25.8)	11 (27.5)	22 (25)	0.77
Anterior chest pain, *n* (%)	66 (51.6)	22 (55)	44 (50)	0.60
Posterior chest pain or lumbar pain, *n* (%)	48 (37.5)	18 (45)	30 (34.1)	0.24
Abdominal pain, *n* (%)	31 (24.2)	10 (25)	21 (23.9)	0.89
Syncope, *n* (%)	11 (8.6)	6 (15)	5 (5.7)	0.082
Perfusion deficit, *n* (%)	17 (13.3)	7 (17.5)	10 (11.4)	0.34
Time from symptom onset (hours), median (IQR) [*n* = 99]	10 (2–48)	3 (1–13)	12 (2–72)	0.085
Systolic blood pressure (mmHg), median (IQR)	135 (120–150)	135 (120–150)	138 (125–150)	0.19
Diastolic blood pressure (mmHg), median (IQR)	80 (70–80)	70 (60–80)	80 (70–83.5)	0.005
Heart rate (bpm), median (IQR)	74 (65–85)	80 (67–90)	74 (64–80)	0.12
Hypertension, *n* (%)	99 (77.3)	30 (75)	69 (78.4)	0.67
Diabetes, *n* (%)	10 (7.8)	2 (5)	8 (9.1)	0.43
Smoke, *n* (%)	18 (14.1)	6 (15)	12 (13.6)	0.84
Drugs, *n* (%) [*n* = 111]	2 (1.6)	0 (0)	2 (2.3)	0.37
CAD, *n* (%) [*n* = 111]	14 (10.9)	3 (9.7)	11 (12.5)	0.56
Previous surgery for AAS, *n* (%) [*n* = 93]	67 (72)	19 (79.2)	48 (69.6)	0.37
Previous TEVAR for AAS, *n* (%) [*n* = 50]	22 (44)	9 (47.4)	13 (41.9)	0.71
White blood cell count, ×10^3^/µL [*n* = 94]	7.71 (6.64–9.71)	8.8 (7.62–10.38)	7.33 (6.38–9.44)	0.027
Creatinine, mg/dL [*n* = 106]	0.99 (0.86–1.25)	1.01 (0.86–1.21)	0.99 (0.86–1.27)	0.98
C-reactive protein, mg/L [*n* = 60]	12.5 (1.8–40.7)	25.1 (11.7–59.2)	7.1 (0.9–32.2)	0.033

Square brackets indicate the number of patients with available data. AltD: alternative diagnosis; CAD: coronary artery disease; IQR: interquartile range; nAAS: novel acute aortic syndrome.

**Table 2:** Prevalence of clinical variables used for pre-test probability assessment, and patient stratification according to the ADD and AORTAs scores.

	All Patients (*n* = 128)	nAAS (*n* = 40)	AltD (*n* = 88)	Odds Ratio	*p*-Value
Marfan/other connective tissue disease, *n* (%)	11 (8.6)	3 (7.5)	8 (9.1)	0.81 (0.2–3.23)	0.77
Family history of acute aortic syndrome, *n* (%)	3 (2.3)	1 (2.5)	2 (2.3)	1.1 (0.1–12.53)	0.94
Known aortic valve disease, *n* (%)	25 (19.5)	12 (30)	13 (14.8)	2.47 (1.01–6.06)	0.045
Recent aortic manipulation, *n* (%)	24 (18.9)	9 (22.5)	15 (17)	1.41 (0.56–3.57)	0.47
Known thoracic aortic aneurism, *n* (%)	16 (12.5)	9 (22.5)	7 (8)	3.36 (1.15–9.8)	0.022
Severe pain, *n* (%)	32 (25)	16 (40)	16 (18.2)	3 (1.3–6.9)	0.009
Sudden pain, *n* (%)	28 (21.9)	17 (42.5)	11 (12.5)	5.17 (2.12–12.6)	<0.001
Ripping pain, *n* (%)	3 (2.4)	3 (7.5)	0 (0)	16.52 (0.83–327.77)	0.010
Pulse asymmetry or systolic blood pressure differential > 20 mmHg, *n* (%)	19 (14.8)	6 (15)	13 (14.8)	1.02 (0.36–2.91)	0.97
Focal neurological deficit, *n* (%)	12 (9.4)	5 (12.5)	7 (8)	1.65 (0.49–5.57)	0.42
New or unknown diastolic aortic murmur, *n* (%)	3 (2.3)	2 (5)	1 (1.1)	4.58 (0.4–52.04)	0.18
Hypotension or shock state, *n* (%)	9 (7)	7 (17.5)	2 (2.3)	9.12 (1.8–46.18)	0.002
ADD score = 0	34 (26.6)	6 (15)	28 (31.8)		
ADD score = 1, *n* (%)	53 (41.4)	8 (20)	45 (51.1)		
ADD score ≥ 2, *n* (%)	41 (32)	26 (65)	15 (17)		
AORTAs score = 0	57 (44.5)	10 (25)	47 (53.4)		
AORTAs score = 1, *n* (%)	41 (32)	11 (27.5)	30 (34.1)		
AORTAs score ≥ 2, *n* (%)	30 (33.4)	19 (47.5)	11 (12.5)		

AltD: alternative diagnosis; nAAS: novel acute aortic syndrome.

**Table 3:** Diagnostic accuracy of ADD and AORTAs score for diagnosis of novel acute aortic syndrome in study patients.

	TP	FP	TN	FN	Sensitivity (%)	Specificity (%)	LR+	LR−
ADD ≥ 1	34	60	28	6	85 (70.2–94.3)	31.8 (22.3–42.6)	1.25 (1.03–1.51)	0.47 (0.21–1.05)
ADD ≥ 2	26	15	73	14	65 (48.3–79.4)	83 (73.4–90.1)	3.81 (2.28–6.38)	0.42 (0.27–0.65)
AORTAs ≥ 1	30	41	47	10	75 (58.8–87.3)	53.4 (42.5–64.1)	1.61 (1.21–2.14)	0.47 (0.26–0.83)
AORTAs ≥ 2	19	11	77	21	47.5 (31.5–63.9)	87.5 (78.7–93.6)	3.8 (2–7.22)	0.6 (0.44–0.81)

**Table 4:** Diagnostic accuracy of d-dimer for diagnosis of novel acute aortic syndrome, in study patients.

	TP	FP	TN	FN	Sensitivity (%)	Specificity (%)	LR+	LR−
≥500 ng/mL	35	60	8	0	100 (90–100)	11.8 (5.2–21.9)	1.13 (1.04–1.24)	0
≥750 ng/mL	35	57	11	0	100 (90–100)	16.2 (8.4–27.1)	1.19 (1.07–1.32)	0
≥1000 ng/mL	29	57	11	6	82.9 (66.4–93.4)	23.5 (14.1–35.4)	1.08 (0.89–1.32)	0.73 (0.31–1.7)
≥age-adjusted cutoff *	35	59	9	0	100 (90–100)	13.2 (6.2–23.6)	1.15 (1.05–1.26)	0

* Age-adjusted cutoff was calculated as patient age ×10, with a minimum value of 500 ng/mL; LR: likelihood ratio (+: positive; −: negative).

**Table 5:** Diagnostic performance of strategies integrating clinical score with d-dimer for rule-out of novel acute aortic syndrome in study patients.

	N	FN	Sensitivity (%)	Specificity (%)	LR−	Efficiency *
ADD = 0 or DD < 500 ng/mL	2	0	100 (90–100)	2.9 (0.4–10.2)	0	1 in 52
ADD = 0 or DD < 1000 ng/mL	5	0	100 (90–100)	7.4 (2.4–16.3)	0	1 in 21
ADD ≤1 or DD < 500 ng/mL	5	0	100 (90–100)	7.4 (2.4–16.3)	0	1 in 21
ADD ≤1 or DD < 1000 ng/mL	13	2	94.3 (80.8–99.3)	16.2 (8.4–27.1)	0.35 (0.08–1.51)	1 in 8
AORTAs = 0 or DD < age-adj. cutoff *	4	0	100 (90–100)	5.9 (1.6–14.4)	0	1 in 26
AORTAs = 0 or DD < 1000 ng/mL	8	1	97.1 (85.1–99.9)	10.3 (4.2–20.1)	0.28 (0.04–2.17)	1 in 13
AORTAs ≤ 1 or DD < age-adj. cutoff *	8	0	100 (90–100)	11.8 (5.2–21.9)	0	1 in 13
AORTAs ≤ 1 or DD < 1000 ng/mL	17	5	85.7 (69.7–95.2)	17.7 (9.5–28.8)	0.81 (0.31–2.11)	1 in 6

DD: d-dimer; FN: number of false negative cases; LR−: negative likelihood ratio; N: number of patients satisfying rule-out criteria. * calculated as proportion of patients satisfying rule-out criteria amongst tested patients.

## Data Availability

The data presented in this study are available on reasonable request from the corresponding author. The data are not publicly available, since authorization of data storage in a public repository was not obtained by the Ethics Committee.
